# Urban food insecurity in the context of high food prices: a community based cross sectional study in Addis Ababa, Ethiopia

**DOI:** 10.1186/1471-2458-14-680

**Published:** 2014-07-04

**Authors:** Tesfay Birhane, Solomon Shiferaw, Seifu Hagos, Katia Sarla Mohindra

**Affiliations:** 1 College of Health Science, Debre Berhan University, P.O. Box 445, Debre Birhan, Ethiopia; 2 School of Public Health, College of Health Sciences, Addis Ababa University, Addis Ababa, Ethiopia; 3 Globalization/Health Equity Unit, University of Ottawa, Ottawa, Canada

**Keywords:** Food insecurity, Food price, Urban, Ethiopia

## Abstract

**Background:**

High food prices have emerged as a major global challenge, especially for poor and urban households in low-income countries such as Ethiopia. However, there is little empirical evidence on urban food security and how people living in urban areas are coping with sustained high food prices. This study aims to address this gap by investigating the food insecurity situation in urban Ethiopia -a country experiencing sustained high food prices, high rates of urban poverty, and a growing urban population.

**Methods:**

A community based cross-sectional study was conducted from January 18 to February 14, 2012. A total of 550 households were selected from three sub-cities of Addis Ababa using three-stage sampling technique. Data were collected using questionnaire based interview with household heads. Items in the questionnaire include, among others, basic socioeconomic variables, dietary diversity and coping strategies. Food security status of households was assessed by a Household Food Insecurity Access Score. Data analysis was done using SPSS software and both univariate and bivariate analysis were done.

**Results:**

The study found that 75% of households were food insecure and 23% were in a state of hunger. Households with higher food insecurity scores tend to have lower dietary diversity and are less likely to consume high quality diets. Reduction in meal size and shifting to poor quality/less expensive/food types were among the common coping strategies to high food price used by households. Household incomes, occupational and educational status of household heads were significant determinants of food security.

**Conclusion:**

Food insecurity in Ethiopia is not only a rural problem. Urban food insecurity is a growing concern due to the toxic combination of high rates of urban poverty, high dependency of urban households on food supplied by the market, and fluctuating food prices. Household food insecurity was particularly high among low income households and those headed by uneducated, daily wagers and government employed household heads. Therefore, policy makers should work on stabilization of the food market and creating opportunities that could improve the livelihood and purchasing power of urban households.

## Background

Ethiopia, a country that has a lengthy history of challenges linked to rural food security, is currently facing relatively new challenges related to urban food insecurity. According to the Interim Report on Poverty Analysis Study (2010/11), the proportion of the population below the poverty line in urban areas was 25.7%, while the proportion of ‘food poor’ people (people who could not purchase the consumption items that generate 2, 200 kilo calories) in urban Ethiopia was estimated to be 27.9% in 2011 [1]. The majority of urban households in Ethiopia (over 80%) are dependent on markets for their food supply [2].

Since August 2004, the Ethiopian food price index has been consistently higher than the world index [3]. According to the Central Statistical Agency, year-on-year food inflation in February 2012 increased by 47.4% compared to February 2011, while non-food inflation increased by 21.4% within the same period. In February 2012, increases were observed in the prices of cereals, pulses, vegetables, fruits and spices [4]. In large metropolitan areas such as Addis Ababa, household food security is being threatened by a combination of forces: a predominantly market-based food supply, persistent chronic poverty, and rising food prices.

Food security is a complex issue that has been defined in a variety of ways. We follow the Declaration on World Food Security, which states that food security exists ‘when all people at all times, have physical and economic access to sufficient, safe and nutritious food to meet their dietary needs and food preferences for an active and healthy life’ [5]. One approach to measuring food insecurity is using the Household Food Insecurity Access Scale (HFIAS), which was used in two recent surveys in Ethiopia. A baseline national food security survey in 2009 by the Ethiopian Health and Nutrition Research institute (EHNRI) reported that 35 percent of households in Ethiopia were food insecure [6], while the magnitude of food insecurity among volunteer AIDS care givers in Addis Ababa in 2009 was about 81% [7]. Hence, there is a need for a broader understanding of food security in metropolitan areas in Ethiopia, including how households use different strategies to cope with poor food access such as reducing the quality, number of meals and the amount of food they consumed, and identifying the determinants of food insecurity, which has been done in urban settings in other low-income countries [8-11]. In this study, we aim to contribute to the understanding of food security in Ethiopia by investigating the level of household food insecurity, its determinants and households coping strategies in Addis Ababa.

## Methods

### Study period and setting

This study was conducted in Addis Ababa from January 18 to February 14, 2012 (which is the period following the harvest season as the majority of crop and food staples are harvested from November to the end of December). Addis Ababa, an important diplomatic capital in Africa, is one of the fastest growing cities on the continent. According to projections based on the 2007 national census for July 2012, the city has a total population of about 3,040,740, which is about 30% of the urban population in Ethiopia. Addis Ababa has a total area of 527 km^2^ and a population density of 5,165.1/km^2^. There are 10 administrative sub-cities and 116 districts [12]. There is a high rate of unemployment (31%) and a large concentration of slum dwellings (over 80%), with the average household size at 4.1 persons, generally living in houses with inadequate sanitation facilities [13].

### Study design and sampling

A community based cross-sectional study was used. The sample size of 550 households was estimated using the formula for single population proportion with the following assumptions: household food insecurity at 35% [6], 95% confidence level, 5% margin of error, 5% allowance for possible non response and design effect of 1.5.

To sample households, a multi-stage (three step) procedure was used: First, the ten administrative sub-cities were stratified into three groups based on social indicators of development (mainly unemployment rate and percentage of households without latrine. Second, one sub-city was selected from each strata using the developmental score given in each group as least, medium and most developed. Finally, after distributing the sample to the three strata based on size of the population, every 6^th^ households were selected using systematic sampling technique using a list of households.

### Survey instrument, procedures and quality assurance

The household food security level was determined using a standardized set of questions derived from version 3 of the Household Food Insecurity Access Scale (HFIAS) measurement guide [14] which was validated in earlier studies [6,7]. The 12 food groups recommended by FANTA using a 24-hour recall method were also used to assess the dietary diversity score of households [15]. Household Dietary diversity, which identifies the diversity and quality of diet consumed by the household, is a measurement tool of food security that can be used together with the HFIAS [15-17]. Data were also collected on household coping strategies during a period of food crisis, causes of household food insecurity, household asset possession, household income and expenditure, and other socio-demographic characteristics.

The original questionnaire was prepared in English (annexed as Additional file 1), then translated into the local language (Amharic) and back-translated into English in order to check for consistency. It was pretested among 30 households outside of the study areas. Six interviewers were recruited and trained for this study. The respondent of the questionnaire was either the household head or care giver of the family. The interview was conducted at the respondent’s house. The principal investigator supervised all activities during the data collection to ensure data quality.

### Study variables

**Figure 1:**
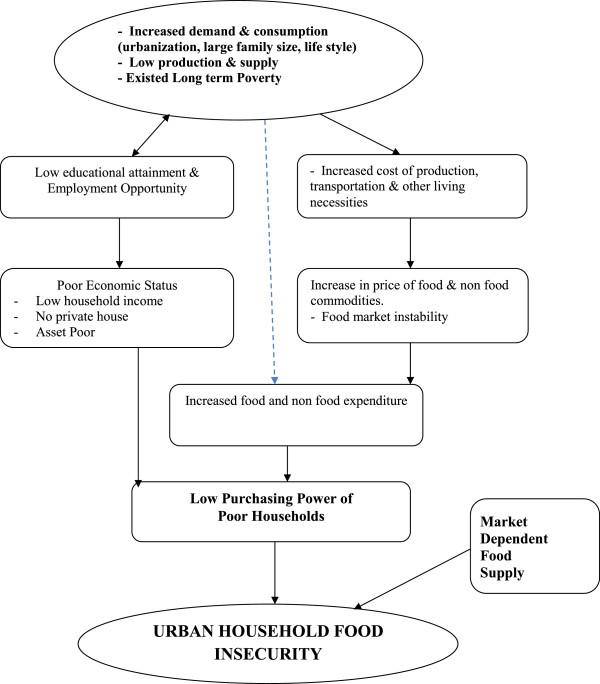
Conceptual framework of urban Household food Insecurity by Access: Addis Ababa, January 2012.

The outcome variable was the level of household food insecurity. Based on the response to the nine HFIAS questions and their frequency of occurrence over the past 30 days, households were assigned a score between 0 to 27. A higher HFIAS is indicative of poorer access to food and greater household food insecurity [14]. Definition of each food category (secure or mild to severe food insecure) is included under the operational definition (Additional file 2). To identify potential household determinants of food security, demographic and socio-economic characteristics of households were included in the analysis (Figure 1).

### Data processing and analysis

Data were entered into pre-formed Epi-info template and cleaned using frequencies and crosstabs. The dataset was then exported to SPSS version 16 for analysis. Data analysis proceeded in two steps. First, the level of food insecurity across the population was assessed using descriptive analysis. Second, multiple logistic regression analysis was performed to identify determinants of household food security. Associations were established using adjusted odds ratios as a measure of strength of association (with a 95% confidence interval).

### Ethics

Ethical clearance was obtained from the Research Ethics Committee at the School of Public Health, College of Health Sciences, Addis Ababa University. Additional letter of support was obtained from the Addis Ababa City Administration and disseminated to all respective sub-cities. Informed consent was received from the participants and when both the household head and spouse were available during the visit, permission was asked from both parents.

## Results

### Sample characteristics

A total of 550 households participated in the study of which 218 (39.6%) households were female headed. The mean and median age of household head were 45 and 44 years respectively. The average family size was 5, with a range of 1 to 15. A total of 114 (20.7%) household heads were unemployed (Table 1).

**Table 1:** Socio demographic and economic characteristics of study participants Addis Ababa, February, 2012 (n = 550)

**Variables**	**Frequency (n = 550)**	**Percent**
**Sex of HH head**		
Male	332	60.4
Female	218	39.6
Age of HH head*		
18-24	17	3.1
25-44	259	47.1
45-64	195	35.5
65+	79	14.4
**Family size**		
1-4	228	41.5
5-8	276	50.2
9	46	8.3
**Education of HH head**		
Uneducated	146	26.5
Primary school	176	32.0
Secondary school	127	23.1
Diploma & above	101	18.4
**Occupation of HH head**		
Unemployed**	114	20.7
Merchant	48	8.7
Government employed	91	16.5
NGO employed	86	15.6
Self employed	94	17.1
Daily wage	52	9.5
Pension	65	11.8
**Housing ownership**		
Private	134	24.4
Kebele’s rent	217	39.5
Private rent	147	26.7
Gift***	32	5.8
Government rental Housing	20	3.6
**Average monthly income ($) (n = 430)**		
<33.3	114	26.5
33.3-112.1	235	54.7
112.1	81	18.8
**Food share of expenditure (n = 471)**		
<50%	166	35.2
50-75%	228	48.4
>75%	77	16.3

*-*UN-lowest age classification for household earning and employment activities.*

**-Includes those who get remittance as source of income.

***-Gift houses are those given from relatives, NGOs and government for peoples’ shelter.

HH = Household, $ = US dollar classified as percentiles.

### Household income and expenditures

The mean reported monthly household income was 1477.8 Ethiopian birr (82.8 USD). The mean monthly per capita income for this sample was 342 birr (19.2 USD), which is equivalent to 0.64 US dollar per day. The monthly average expenditure of households was 1634 birr (91.6 USD). All of the households sampled in this study purchased their food from the market. Food assumed the largest share of the majority of household consumption expenditure; the mean share of food expenditure was 53.9% and ranged from 0% among destitute households to 95% among pensioner headed households.

### Household access to food, dietary diversity and coping strategies

**Figure 2:**
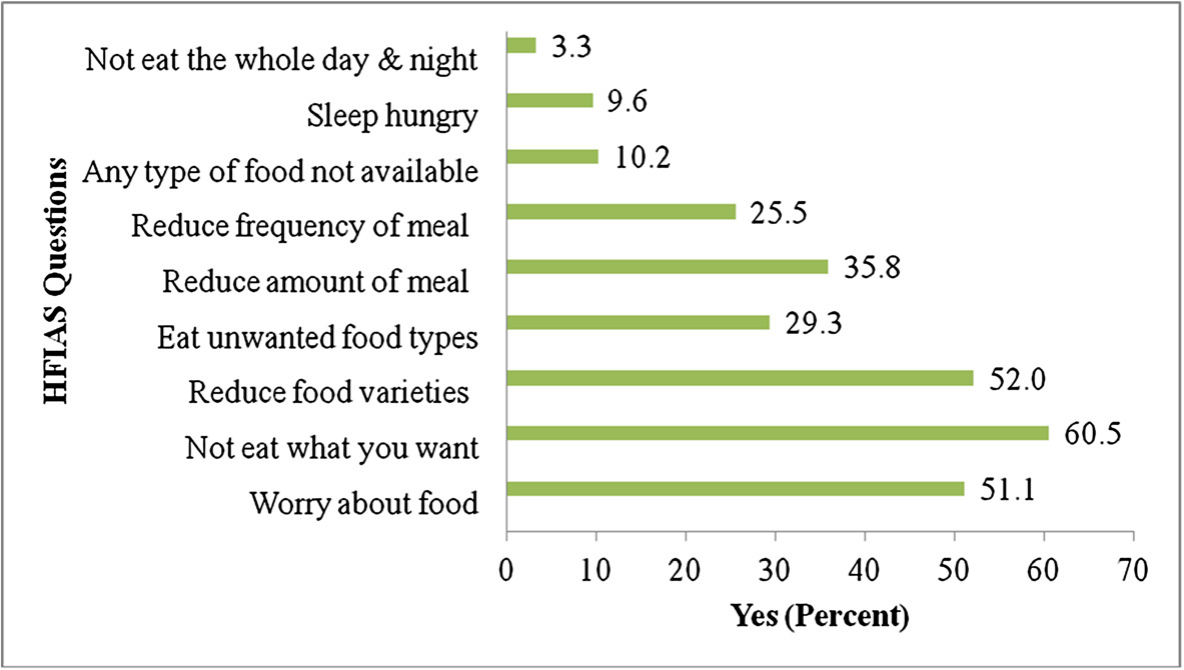
Household Response to HFIAS questions, Addis Ababa, February, 2012 (n = 550).

The mean HFIAS score of the sample households was 5.4. A total of 129 (23.5%) households had a score of 0, indicating they never experienced any form of food insecurity, 53 (9.6%) respondents reported that they have ever experienced sleeping hungry, and 18 (3.3%) participants reported that they did not eat for an entire day at the time of survey. Two hundred eighty six (52%) households have reduced the variety of food that they consumed, 197 (35.8%) have reduced the amount of food that they consume, and 140 (25.5%) have reduced their meal frequency (Figure 2).

**Figure 3:**
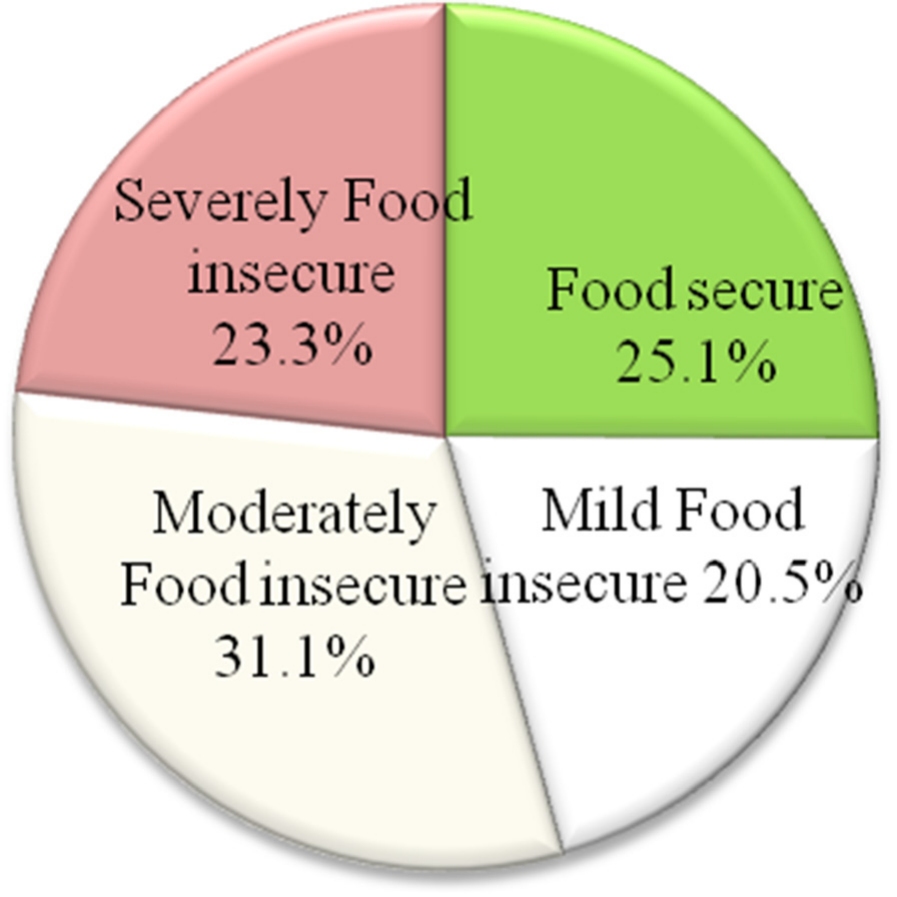
Diagrammatic representation of Food security status in Addis Ababa, February, 2012 (n = 550).

Among the total 550 households, 412 (74.9%) reported scores that classified them as food insecure. According to the scale, 128 (23.3%) of households were classified as severely food insecure, while 113 (20.5%) and 171(31.1%) households were mild and moderately food insecure respectively (Figure 3).

Furthermore, 393 (71.5%) of households perceived that they were experiencing food insecurity and were not able to feed their family properly. The main reasons reported for inadequate food consumption was food shortages due to high food price (65%) and reduction in household income (32.7%), and few households mentioning other reasons, such as loss of appetite and lack of time to prepare.

**Figure 4:**
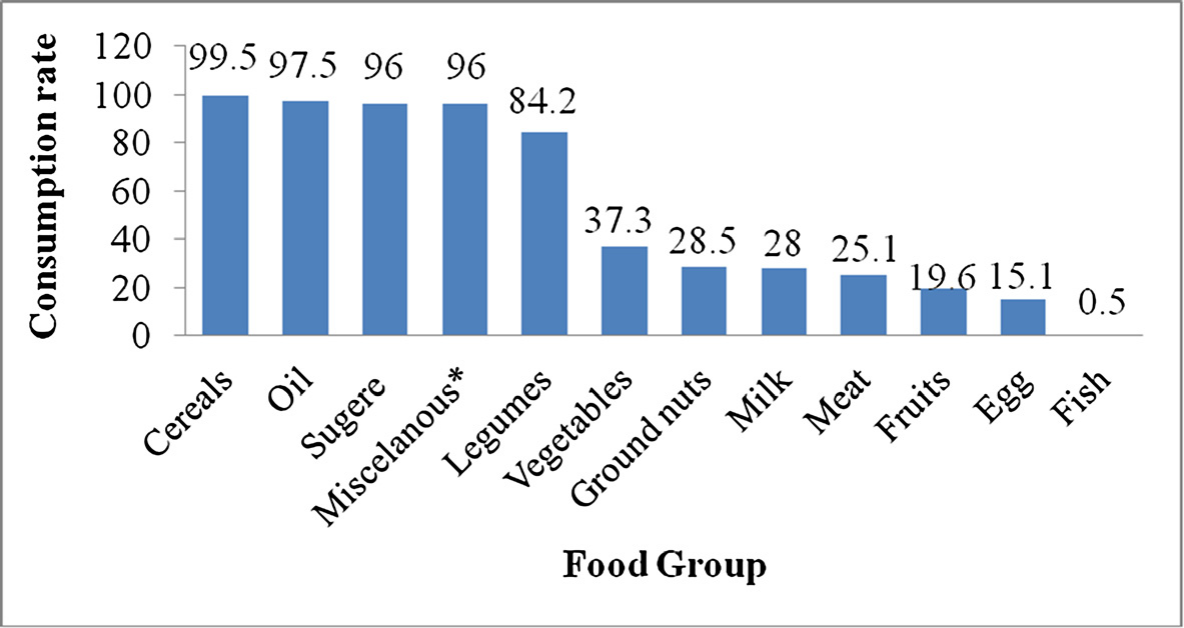
Households’ Consumption rate of different food groups, Addis Ababa, February 2012 (n = 550).

The mean (+SD) dietary diversity score of households was 6.3 ± 0.07. Using this mean score, households were categorized into three equal parts (terciles): 224 (40.7%) of households had consumed 5 or less food groups (poor dietary diversity) while 216 (39.3%) had consumed 7 or more food groups (high dietary diversity). Cereals (99.5%), sugar (96.0%), and miscellaneous foods like tea and coffee (96%) were the most commonly consumed food groups, while fish (0.5%), egg (15.0%) and fruits (19.6%) were the least consumed food groups (Figure 4).

**Figure 5:**
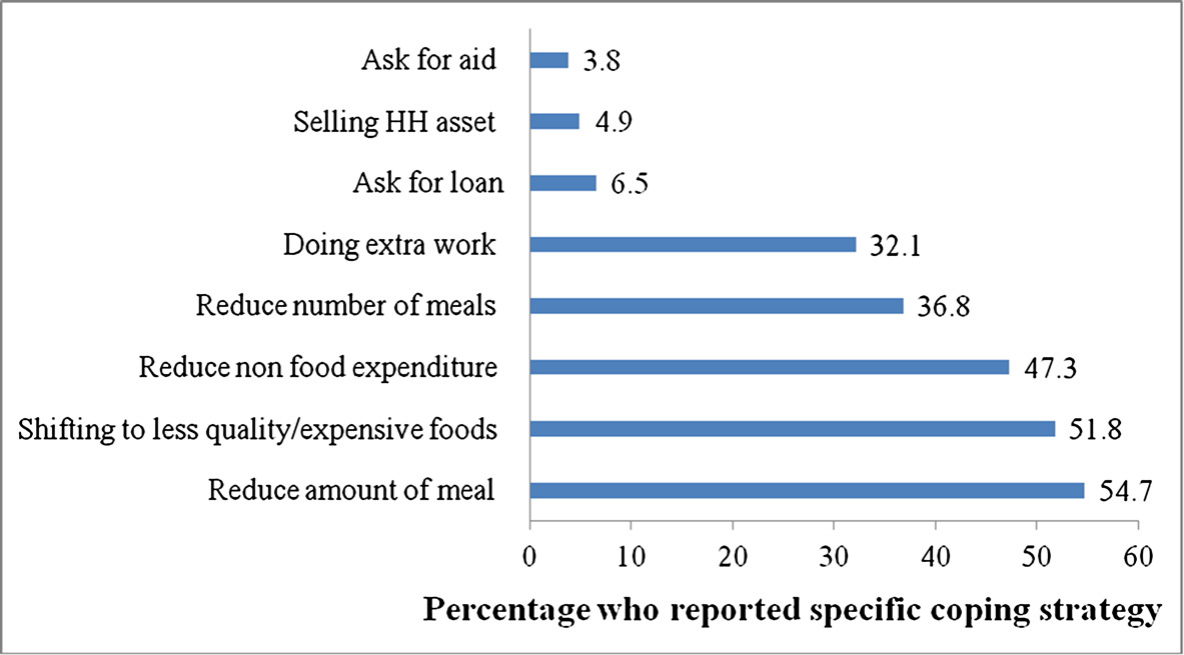
Household coping strategies of high food price and food insecurity, Addis Ababa, February, 2012 (n = 550).

Households used a range of coping strategies when faced with rising food prices and food insecurity, including reducing the amount of meal (54.7%), shifting to less quality/expensive foods (51.8%), skipping meals (36.8%), and reducing non food expenditure (47.3%) (Figure 5).

### Determinants of household food insecurity

The binary logistic regression analysis shows that, household income, asset possession, house ownership, educational and employment status of household heads, and family size are factors associated with food security status. The results of multiple binary logistic regression models indicated that having a lower monthly income (AOR = 3.8, 95% CI = 1.5-9.7) was independently associated with food insecurity. In addition, household heads who were uneducated (AOR = 3.4, 95% CI = 1.6-6.8) daily laborers (AOR = 2.96, 95% CI = 1.1-8.3), and government employees (AOR = 2.3, 95% CI = 1.1-4.9) were more likely to have higher food insecurity. On the other hand those households living in government rental houses were less likely to be food insecure, compared to other residential houses (AOR = 0.34, 95% CI = 0.1-0.95). Sex and age of household head and family size did not show significant associations with household food security status (Table 2).

**Table 2:** Multivariate regression analysis for selected household characteristics and wealth status of households, Addis Ababa 2012

**Variables (n = 550)**	**Food security status [Frequency (%)]**		**COR (95% CI)**	**AOR (95% CI)**
	***Food insecure***	***Food secured***		
**Education of HH head**				
Uneducated	116 (79.5)	30 (20.5)	2.75 (1.57-4.8)	3.4 (1.7-6.7)*
1° School	139 (79)	37 (21.0)	2.67 (1.56-4.57)	2.8 (1.5-5.4)*
2° School	98 (77.2)	29 (22.8)	2.41 (1.36-4.27)	2.2 (1.1-4.1)*
Diploma/above	59 (58.4)	*42 (41.6)*	1.00	1.00
**Occupation of HH head**				
Unemployed^a^	83 (72.8)	31 (27.2)	1.00	1.00
Merchant	27 (56.2)	21(43.8)	0.48 (0.24-0.97)	0.5 (0.3-1.1)
Government employee	69 (75.8)	22 (24.2)	1.17 (0.62-2. 21)	2.3 (1.1-4.9)*
NGO employed	62 (72.1)	24 (27.9)	0.97 (0.52-1.81)	1.3 (0.6-2.6)
Self employed	71 (75.5)	23 (24.5)	1.15 (0.62-2.16)	1.4 (0.7-2.6)
Daily wage	47 (90.4)	5 (9.6)	3.5 (1.3-9.64)	2.96 (1.1-8.3)*
Pension	53 (81.5)	12 (18.5)	1.65 (0.78-3.5)	1.8 (0.9-4.0)
**Housing ownership**				
Private	93 (69.4)	41 (30.6)	1.00	1.00
Kebele rent	174 (80.2)	43 (19.8)	1.78 (1.1-2.9)	1.2 (0.7-2.1)
Private rent	115 (78.2)	32 (21.8)	1.58 (0.93-2.71)	1.3 (0.72-2.5)
Gift^b^	22 (68.8)	10 (31.2)	0.97 (0.42-2.23)	0.99 (0.4-2.41)
Government RH^c^	8 (40)	12 (60.0)	0.29 (0.11-0.78)	0.34 (0.1-0.95)*
**Asset possession**				
Asset poor	197 (87.9)	27 (12.1)	5.4 (3.34-8.79)*	1.4 (0.7-2.5)
Asset medium	91 (82.7)	19 (17.3)	3.55 (2.02-6.24)*	1.3 (0.8-2.1)
Asset rich	124 (57.4)	92 (42.6)	1.00	1.00
**Average monthly income**^d^				
1^st^terciles	99 (86.8)	15 (13.2)	6.8 (3.4-13.6)	3.8 (1.5-9.7)*
2^nd^terciles	183 (77.9)	52 (22.1)	3.61(2.12-6.15)	2.4 (1.2-4.9)*
3^rd^terciles	40 (49.4)	41 (50.6)	1.00	1.00

*-Significant association at 95% CI and p < 0.05 when adjusted for other variables.

^*a*^*-Includes those who get remittance as source of income.*

^*b*^*-Households supported by relatives, charities or government.*

^*c*^*-RH = rental house.*

^*d*^*-Analysis is done for 430 Households because of incomplete data on income.*

HH = Household, COR = Crude odds ratio, AOR = adjusted odds ratio, CI = confidence interval.

## Discussion

This study demonstrated that there is a high level of (74.9%) food insecurity in Addis Ababa. The results are considerably higher than the national food insecurity (35%) reported by the Ethiopian Health and Nutrition Research Institute in 2009 [6]. The higher level of food insecurity identified in our study, could be due to the high food inflation in Addis Ababa during the past two years, contributing to greater food insecurity. Another explanation is that this study was specific to a large metropolitan area, where households are dependent on markets for their food supply.

The food diversity score was similar to what was found in another study undertaken in Addis Ababa and in Kenya [7,16]. Cereals were the most commonly consumed food groups by the study population, with little consumption of fruits, vegetables, and animal products, which can contribute to malnutrition and higher diseases burdens [18].

Reducing the size of meals and shifting to poorer quality or less expensive foods were commonly reported household coping strategies of food insecurity which is consistent with findings from Bangladesh [8]. Beside its psychological effect, these coping mechanisms are indications of under nutrition [19] and can induce other nutritional problems like obesity [20].

Household income was a major determinant of food insecurity. Households headed by individuals who were unemployed and had less educational attainment are also more likely to be food insecure. This is consistent with other studies [9-11]. Uneducated heads are less likely to be employed, especially in the context of the present global economic crisis. We did not find any significant differences between male and female-headed households with respect to household food insecurity status, which is similar to what studies have found in other low-income countries [10,11]. This may be due to the widespread nature of food insecurity; a more in-depth understanding of food insecurity and gender would require further study, potentially using comparative studies and qualitative approaches.

Since this study was conducted during the post harvest time, this finding reveals that poor urban households, unlike their rural counterparts, remain food insecure even after the harvest of crops, which may be due to a rise in food prices. Although we did not evaluate causal relationship between food price and food insecurity, the majority of households reported high food prices as the main reason for food insecurity. This suggests that policy makers view urban and rural food insecurity as different but interlinked problems.

There are three main limitations to this study. First, the design was cross-sectional; therefore, we were unable to investigate causal links between rising food prices and urban food insecurity. Second, a cross-sectional design enables us only to assess food security at one point of the harvest cycle, precluding an understanding of food security across all of the seasons. Third, the HFIAS questions can be influenced by social desirability bias though attempt was made to minimize it by clarifying the purpose of the study.

## Conclusion

Food insecurity in Ethiopia is not only a rural problem. Urban food insecurity is a growing concern due to the toxic combination of high rates of urban poverty, high dependency of urban households on food supplied by the market, and fluctuating food prices. Households in Addis Ababa, not only do not have a sufficient amount of food to eat, their diets are largely cereal based, lacking an adequate diversity of food to yield good nutrition. Subsidization of common food commodities should be strengthened to improve access to the poor. Emphasis should be given to household heads engaged as daily wage earners, public employees, and to those who have little to no education.

We recommend three main avenues for further research. First, there is a need for longitudinal studies that will enable an assessment of seasonality in food insecurity in an urban context and to better understand the relationship between rising food prices and food insecurity. Second, there is a need for more research that adopts an equity lens in order to be able to identify specific groups that are especially vulnerable to rising food prices. This might include in-depth studies of intra-household dynamics in relation to food security. Finally, given the widespread nature of food insecurity and the volatility of global and local food markets, there is a need to undertake research that will inform best practices for increasing food security among urban populations.

## Competing interests

The authors declare that they have no competing interests.

## Authors’ contributions

SS conceived the idea. TB designed the study, coordinated the data collection, entered and analyzed data and wrote the first draft of the manuscript. SS and SH critically reviewed the research proposal and final result and all authors contributed to the interpretation of results. KM provided input on interpretations of study findings and reviewed drafts of the manuscript. All authors read and approved the final manuscript.

## Pre-publication history

The pre-publication history for this paper can be accessed here:

http://www.biomedcentral.com/1471-2458/14/680/prepub

## Supplementary Material

Additional file 1 English Version questionnaires to assess household food insecurity in the context of high food prices in Addis Ababa, February, 2012. Click here for file

Additional file 2 Operational definition for household food insecurity in the context of high food prices in Addis Ababa, February, 2012. Click here for file
