# Supplementary material for: Urban food insecurity in the context of high food prices: a community based cross sectional study in Addis Ababa, Ethiopia
Source: BMC Public Health. 2014 Jul 4;14:680. doi: 10.1186/1471-2458-14-680 (PMC4227136; doi:10.1186/1471-2458-14-680)
Supplement: Additional file 1 — English Version questionnaires to assess household food insecurity in the context of high food prices in Addis Ababa, February, 2012. [file 1471-2458-14-680-S1.docx]

# Annex II: English Version questionnaires

**Questionnaires to assess household food insecurity in the context of high food prices in Addis Ababa, February, 2012**

Questionnaire serial number (code): _______ Date of data collection: ________

Sub-City________ Wereda _______ House hold number: _________

Data collector’s **Name:** ______________________ **Signature:** _____

Supervisor‘s **Name:** _________________________ **Signature:** _____

**Section 1: Questions about general households’ Economic and demographic characteristics**

| **S. No** | **Questions** | **Respond options** | **Code** |
| --- | --- | --- | --- |
|  | Sex of Household head | 1. Male 2. Female | \|___\| |
|  | What is the Age of Household head | 1. ________________years | \|___\| |
|  | Religion | 1. Orthodox 2. Protestant 3. Muslim 4. Catholic 5. Others __ | \|___\| |
|  | What is the educational status of household head | 1. Uneducated 2. Elementary school 3. Secondary school   4. Diploma and above | \|___\| |
|  | What is the family size of your Household | ______________ | \|___\| |
|  | Who is the owner of the house you live in? | 1. It is our private house 2. Gifted from government /relatives/ 3. Kebele rent 4. Rent from private owner |  |
|  | What is the employment status of household head | 1. Unemployed 2. Self employed 3. Government (NGO) employed 4. Wage labor 5. Pensioner 6. Other (specify):__________ | \|___\| |
|  | Average monthly house hold income (*put the sum of incomes contributed by family members*) | ____________Birr |  |
|  | What is the average monthly expenditure of your Household | ____________Birr |  |
|  | In the last six months what was the main source of your household food consumption | 1. Purchased from market 2. From own production 3. Remittance from any agent 4. Begging 5. Others (specify)___________ | \|___\| |
|  | Could you tell me the share of the following commodities in your household expenditure (*in percentage or Birr)* | 1. Food items _________ 2. House rent _________ 3. Cookingfuel/charcoal/electricity_______ 4. Water and light 5. Education_______ 6. Transport __________ 7. Social ______ 8. Alcohol/other substances 9. Others (specify) ___________ |  |
|  | Could you tell me which the type of Asset available at your house (circle all possible responses and underline on each asset to specify it) | 1. TV, DVD, Radio/Tape, Dish, 2. Refrigerators. 3. Modern Beds, tables and chair 4. Sofa set 5. Shelf 6. Jewelry (gold/silver): necklaces, ring, 7. Bicycle, motorcycle 8. Car |  |
|  | In the last 6 months, have you ever sold any type of HH Asset to cover your expenditure? | 1. No 2. Yes, if yes how many (1, 2, >3) | \|___\| |

**Section 2: Occurrence and Frequency of Household Food Insecurity**

| **S. NO** | | **Question** | | **Response options** | **Code** | |
| --- | --- | --- | --- | --- | --- | --- |
|  | | In the past four weeks, did you ***worry*** that your HH would not have enough food? | | 0 = No (skip to Q2)  1=Yes | \|___\| | |
| 2.1.a | | How often did this happen? | | 1 = Rarely (once or twice in the last 4 weeks )  2 = Sometimes (3-10 times)  3 = Often (more than ten times) | \|___\| | |
|  | | In the past four weeks, were you or any HH member ***not able to eat the kinds of foods you preferred*** because of a lack of resources? | | 0 = No (skip to Q3)  1=Yes | \|___\| | |
| 2.2.a | | How often did this happen? | | 1 = Rarely (once or twice)  2 = Sometimes (3-10 times)  3 = Often (more than ten times) | \|___\| | |
|  | | In the past four weeks, did you or any HH member have to eat ***a limited variety of foods*** due to a lack of resources? | | 0 = No (skip to Q4)  1 = Yes | \|___\| | |
| 2.3.a | | How often did this happen? | | 1 = Rarely (once or twice)  2 = Sometimes (3-10 times)  3 = Often (more than ten times | \|___\| | |
|  | | In the past four weeks, did you or any HH member have to ***eat some foods that you really did not want to eat*** because of a lack of resources to obtain other types of food? | | 0 = No (skip to Q5)  1 = Yes | \|___\| | |
| 2.4.a | | How often did this happen? | | 1 = Rarely (once or twice)  2 = Sometimes (3-10 times)  3 = Often (more than ten times) | \|___\| | |
|  | | In the past four weeks, did you or any HH member have to ***eat a smaller meal than you felt*** you needed because there was not enough food? | | 0 = No (skip to Q6)  1 = Yes | \|___\| | |
| 2.5.a | | How often did this happen? | | 1 = Rarely (once or twice)  2 = Sometimes (3-10 times )  3 = Often (more than ten times) | \|___\| | |
|  | | In the past four weeks, did you or any other HH member have to eat ***fewer meals in a day*** because there was not enough food? | | 0 = No (skip to Q7)  1 = Yes | \|___\| | |
| 2.6.a | | How often did this happen? | | 1 = Rarely (once or twice)  2 = Sometimes (3-10 times)  3 = Often (more than ten times) | \|___\| | |
|  | | In the past four weeks, was there ***ever no food to eat of any kind*** in your HH because of lack of resources to get food? | | 0 = No (skip to Q8)  1 = Yes | \|___\| | |
| 2.7.a | | How often did this happen? | | 1 = Rarely (once or twice)  2 = Sometimes (3-10 times)  3 = Often (more than ten times) | \|___\| | |
|  | | In the past four weeks, did you or any HH member ***go to sleep at night hungry*** because there was not enough food? | | 0 = No (skip to Q9)  1 = Yes | \|___\| | |
| 2.8.a | | How often did this happen? | | 1 = Rarely (once or twice)  2 = Sometimes (3-10 times)  3 = Often (more than ten times) | \|___\| | |
|  | | In the past four weeks, did you or any HH member ***go a whole day and night without eating anything*** because there was not enough food? | | 0 = No (skip to section 3)  1 = Yes | \|___\| | |
| 2.9.a | | How often did this happen? | | 1 = Rarely (once or twice)  2 = Sometimes (3-10 times)  3 = Often (more than ten times) | \|___\| | |
| ***Derived from version 3 of the Household Food Insecurity Access Scale (HFIAS) measurement guide*** | | | | | | |
| 2.10 | In the past 4 weeks, do you believe that all member of your household have feed the type of food they want both in quality and quantity | | 0= no  1= yes (skip to Q 3.3 ) | | | \|___\| |
| 2.11 | If you say no, what do you think is the reason for? Because: | | 1. Of illness and loss of appetite 2. Unable to prepare due to lack of time 3. Food is not available at home and market 4. We couldn’t by food due to increased in price 5. We couldn’t by food due to loss of my income 6. Others (specify):___________ | | | \|___\| |
| 4.12 | Different people take different actions to cope with increase in food price, what about you and your family member did? | | 1. Reduce the amount of consumed diet per meal 2. Cut the number of meals consumed per day 3. Shift to less expensive and poor quality diet 4. Reduce on non food expenditures 5. Receive for food or cash aid 6. Taking a loan from bank or other person 7. Selling any house hold assets 8. Others (specify)__________ | | | \|___\| |

**Section 3: Food diversity & consumption score of Households in Addis Ababa, January 2012**

| Now, I will ask you the different food groups if your household members have consumed in the last 24 hours? You will respond by saying Yes or No for each food group.*( if there is any special diet ceremony in the house, please ask about the day before that day)* | | | | **Code** |
| --- | --- | --- | --- | --- |
| **Questions** | **(1) Yes *(√)*** | **(2) No *(√)*** | |  |
| Cereals (*teff*, *wheat, maiz, rice, sorghum,* & their products like ‘pasta’, Macaroni, porridge |  | |  | \|___\| |
| Pulses/legumes |  | |  | \|___\| |
| Vegetables |  | |  | \|___\| |
| Fruits |  | |  | \|___\| |
| Root and tubers |  | |  | \|___\| |
| Meat, poultry |  | |  | \|___\| |
| Eggs |  | |  | \|___\| |
| Fish and seafood |  | |  | \|___\| |
| Milk and milk products |  | |  | \|___\| |
| Oil/fats |  | |  | \|___\| |
| Sugar/honey |  | |  | \|___\| |
| Miscellaneous (other foods) |  | |  | \|___\| |

I have finished my interview and if you have any question or suggestion you can raise, if not I ask you to put your signature confirming that this data represents you and your household.

I confirm that this data is mine: date ________Signature________
