# Supplementary material for: Urban food insecurity in the context of high food prices: a community based cross sectional study in Addis Ababa, Ethiopia
Source: BMC Public Health. 2014 Jul 4;14:680. doi: 10.1186/1471-2458-14-680 (PMC4227136; doi:10.1186/1471-2458-14-680)
Supplement: Additional file 2 — Operational definition for household food insecurity in the context of high food prices in Addis Ababa, February, 2012. [file 1471-2458-14-680-S2.docx]

## Operational definitions

***Food secure Household***: Household ***experiences none of*** the food insecurity (access) conditions, or just ***experiences worry, but rarely***.

***Mildly food insecure (access) Household***: worries about not having enough food sometimes or often, and/or is unable to eat preferred foods, and/or eats a more monotonous diet than desired and/or some foods considered undesirable, but only rarely. But it does not cut back on quantity nor experience any of three most severe conditions.

***Moderately food insecure Household***: sacrifices quality more frequently, by eating a monotonous diet or undesirable foods sometimes or often, and/or has started to cut back on quantity by reducing the size of meals or number of meals, rarely or sometimes. But it does not experience any of the three most severe conditions.

***A severely food insecure Household:*** has forced to cutting back on meal size or number of meals often, and/or experiences any of the three most severe conditions (running out of food, going to bed hungry, or going a whole day and night without eating), even as infrequently as rarely.

**Dietary diversity:** the number of different foods or food groups consumed over 24 hour period

**Household Asset** is the number and types of Household furniture and asset materials like basic Household possessions such as beds, table and chairs, sofa sets, radios and/or televisions, computers, jewelries, transport assets such as bicycle, motorcycle or car.

**Coping strategies:** Coping strategies are the means people employ to master, tolerate, reduce, or minimize the negative consequences of changes in food price and their food security status.

**Head of Household**: A head of a Household is a person who economically supports or manages the Household or for reasons of age or respect, is considered as head by members of the Household or declares himself as head of a Household.

**Self employed**: This is an individual who works in his own enterprise using his/her own skills and generating his/her Household income without hiring any one. This doesn’t include those merchants.

**Wage/salary**: includes all payments in cash or in kind made to employees in connection with work done for enterprise/establishment or activities.
